# Optimized Method for Quantifying Bisphenols in Bottled Water and PET/rPET Matrices

**DOI:** 10.3390/foods14172968

**Published:** 2025-08-26

**Authors:** Fabiana Di Duca, Paolo Montuori, Elvira De Rosa, Immacolata Russo, Raffaele Palladino, Stefano Scippa, Giuseppe Dadà, Maria Triassi, Sergi Díez

**Affiliations:** 1 Department of Public Health, University “Federico II”, Via Sergio Pansini 5, 80131 Naples, Italy; 2 Department of Human Sciences and Quality of Life Promotion, San Raffaele University, 00166 Rome, Italy; 3 Department of Public Health, University Hospital of Naples “Federico II”, Via Sergio Pansini 5, 80131 Naples, Italy; 4 Interdepartmental Research Center in Healthcare Management and Innovation in Healthcare (CIRMIS), Via Sergio Pansini 5, 80131 Naples, Italy; 5 Department of Primary Care and Public Health, School of Public Health, Imperial College, London SW7 2AZ, UK; 6 CORIPET-Consorzio Volontario per Riciclo del PET, Via S. Maurilio 23, 20123 Milan, Italy; 7 Environmental Chemistry Department, Institute of Environmental Assessment and Water Research, IDAEA-CSIC, C/Jordi Girona, 18-26, E-08034 Barcelona, Spain

**Keywords:** Polyethylene Terephthalate (PET), recycled PET (rPET), Non-Intentionally Added Substances (NIAS), analytical method development, bisphenols

## Abstract

The growing use of plastics in food packaging has raised concerns about chemical migration into consumables, posing potential health risks. Ensuring the safety of packaging materials is a critical public health priority. This study aimed to validate an analytical method for qualitative and quantitative determination of BPs in bottled water and evaluate their presence in PET and rPET matrices. The method was validated through recovery tests for eight BPs (Bisphenol A, Bisphenol S, Bisphenol F, Bisphenol AF, Bisphenol AP, Bisphenol B, Bisphenol Z, and Bisphenol P). Linearity (R^2^ ≥ 0.990) and high recovery rates proved the method’s stability, reliability, and accuracy. For bottled water, LODs ranged 0.030–0.075 µg/L and LOQs 0.10–0.25 µg/L; for PET/rPET, LODs were 0.00030–0.00075 mg/kg and LOQs 0.0010–0.0025 mg/kg. Mean recoveries in bottled water were in the range 89–109%, in PET from 94% to 117%, and in rPET from 106% to 118%. The results showed that BPA was quantifiable in all matrices, while other BPs remained below the limit of quantification. The validated method provides a robust tool for assessing bisphenol contamination and supports ongoing efforts to enhance food safety and inform regulatory frameworks for sustainable PET recycling.

## 1. Introduction

Concerns about the presence of bisphenols (BPs) in polyethylene terephthalate (PET) and recycled polyethylene terephthalate (rPET) matrices have grown significantly in recent years, as their potential impact on human health and the environment has become increasingly apparent, highlighting the urgent need for further research and effective management strategies [1,2].

**Figure 1:**
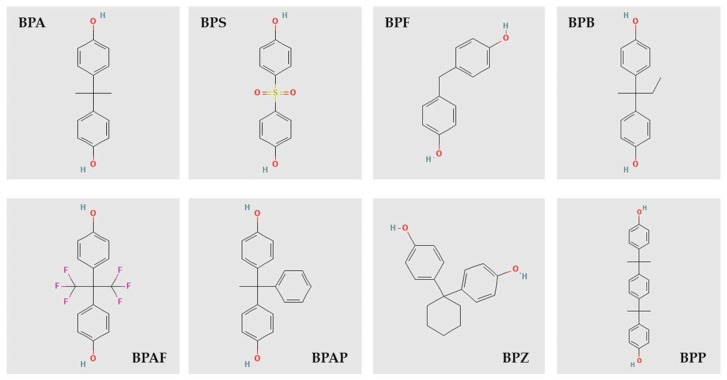
Chemical structures of the compounds investigated in this study.

Bisphenols (BPs) are synthetic organic compounds extensively used in industrial applications because they enhance the durability and heat resistance of polymers [3]. Among these, bisphenol A (BPA) is the most widely studied and is recognized as an endocrine-disrupting chemical (EDC) that can interfere with hormonal signaling, posing potential risks to both human health and the environment [4]. Accordingly, numerous studies have linked BPA exposure to a variety of adverse health effects, including diabetes, obesity, and cardiovascular diseases [5,6,7]. In response to increasing restrictions on BPA in food contact materials (FCMs), alternative BPs such as bisphenol S (BPS), bisphenol F (BPF), bisphenol Z (BPZ), bisphenol AP (BPAP), bisphenol P (BPP), and bisphenol B (BPB) have been introduced as industrial substitutes [8]. However, these compounds share structural similarities with BPA (Figure 1) and raise comparable toxicity concerns, exhibiting similar estrogenic, anti-estrogenic, androgenic, and anti-androgenic activities [9].

Therefore, trace levels of these BPs have been detected in polyethylene terephthalate (PET) and recycled PET (rPET), materials widely used for food and beverage packaging [10]. While BPA in bottled water has been widely studied, its occurrence in PET remains relatively underexplored, likely because BPs are not intentionally used in PET production [2,11]. Nevertheless, BPs can be present in rPET due to contamination from external sources. Potential origins include cross-contamination during recycling processes, where printing inks, adhesives, or coatings from recycled materials may introduce BPs into the recycled PET stream [12]. Furthermore, catalysts or impurities in monomers and additives used during polymerization can contribute to the occurrence of BPs. In recycling processes, contamination often originates from non-PET materials such as epoxy coatings or polycarbonate (PC) plastics, which may release BPs during processing [10]. Although BPs are commonly employed as additives in polycarbonates, they can also occur as non-intentionally added substances (NIAS) in other plastics, including PET and rPET [13]. Environmental contamination during the collection and sorting of recyclables can introduce BPs, which may subsequently migrate into rPET under thermal and mechanical stresses during recycling processes [14]. Even at trace levels, these contaminants raise concerns regarding the safety and quality of rPET, a key material for sustainable packaging. This underscores the need for stringent quality controls and effective decontamination strategies in PET recycling to minimize potential health and safety risks.

In Italy, the legal limit for BPA concentration in drinking water is currently set at 2.5 µg/L, in compliance with Directive (EU) 2020/2184 on the quality of water intended for human consumption [15]. Furthermore, the European Union (EU) and international regulatory frameworks have implemented strict measures to limit BP contamination in plastics. In Europe, Regulation (EU) 10/2011 on plastic materials and articles intended to come into contact with food specifies migration limits for BPA at 0.05 mg/kg [16,17]. However, current regulations do not comprehensively address all BPs, potentially overlooking emerging analogs such as BPS and BPF. Furthermore, rPET intended for food contact applications must comply with Regulation (EU) 2022/1616, which requires thorough safety assessments to ensure that migration limits for contaminants, including BPs, are not exceeded, and mandates effective decontamination processes to minimize associated risks [18]. These measures are designed to protect consumer health and ensure the safety of recycled plastics used in FCMs. Similarly, the U.S. Food and Drug Administration (FDA) imposes strict migration limits for BPA in FCMs but does not fully regulate other BPs. Instead of applying uniform BPA limits across all FCMs, the FDA evaluates its safety based on a reasonable certainty of no harm, considering exposure levels and toxicological risks. These evaluations ensure that BPA migration into food remains within acceptable limits, accounting for cumulative dietary exposure. In cases of negligible exposure, certain uses may qualify for exemptions under the FDA’s food additive regulations [19].

Advanced analytical techniques are crucial for accurately determining BPs in PET and rPET matrices. Among these, high-performance liquid chromatography coupled with tandem mass spectrometry (HPLC-MS/MS) is widely recognized for its exceptional sensitivity, enabling the detection of BPs at ultra-trace levels [10]. Nevertheless, gas chromatography combined with mass spectrometry (GC-MS), after derivatization to improve volatility, remains the most frequently referenced method [20]. Sample preparation typically involves solid-phase extraction (SPE), which is essential for isolating and concentrating BPs from the complex PET and rPET matrices, as well as from water [21,22]. To date, few scientific studies have validated methods for the qualitative and quantitative determination of BPs in rPET matrices, highlighting the need for further research in this area. Most research has focused on migration studies rather than evaluating the concentrations of BPs directly within the matrix, such as in pellets, flakes, or preforms. Furthermore, currently, there is no universally recognized official method for the determination of BPs in the bottled natural mineral water matrix.

Taking the above into account, this study aimed to validate an analytical method for the qualitative and quantitative determination of BPs in bottled water and to evaluate their potential presence in PET and rPET samples derived from mechanical recycling. Specifically, this study involved (i) method validation through recovery tests for eight BPs (Bisphenol A (BPA), Bisphenol S (BPS), Bisphenol F (BPF), Bisphenol B (BPB), Bisphenol AF (BPAF), Bisphenol AP (BPAP), Bisphenol Z (BPZ), Bisphenol P (BPP)) and (ii) the identification and quantification of BPs in PET and mechanically recycled rPET samples, including flakes, granules, and preforms.

## 2. Materials and Methods

### 2.1. Standard Solutions and Reagents

Bisphenol A (BPA, CAS 80-05-7), Bisphenol S (BPS, CAS 80-09-1), Bisphenol F (BPF, CAS 620-92-8), Bisphenol Z (BPZ, CAS 843-55-0), Bisphenol AP (BPAP, CAS 1571-75-1), Bisphenol P (BPP, CAS 2167-51-3), and Bisphenol B (BPB, CAS 1843-11-2) standards were purchased from Sigma-Aldrich (Steinheim, Germany). The process standard used to verify the efficiency of the extraction process was Bisphenol Ad16 (BPAd16) (98% atom D, CAS 96291-66-2), purchased from Sigma-Aldrich (Steinheim, Germany). All reagents had a purity of 99% or higher, ensuring the accuracy and reliability required for the analyses. These high-quality standards enable precise quantification of the target substances in the analyzed samples.

The reagents used included ultrapure water (HPLC-MS grade, Sigma-Aldrich, CAS 7732-18-5), methanol (HPLC-MS grade, Romil, CAS 67-56-1), acetone (HPLC grade, Sigma-Aldrich, CAS 67-64-1), ammonium hydroxide (25% solution, HPLC grade, Sigma-Aldrich, CAS 1336-21-6), formic acid (≥98%, Sigma-Aldrich, CAS 64-18-6), and 1,1,1,3,3,3-hexafluoro-2-propanol (HFIP) (99% purity, Sigma-Aldrich, CAS 920-66-1). Solutions of various compositions were prepared to support different steps of the analysis, including water/methanol (50:50, *v*/*v*) and methanol containing 2% formic acid.

For calibration curves, the standard solutions and the process solution were prepared by dilution of the stock solutions with methanol. In detail, standard solutions were prepared with concentrations in the range 0.10–10.0 µg/L for BPA and BPB and 0.25–10.0 µg/L for BPS, BPF, BPAF, BPAP, BPZ, and BPP.

The list of analytes (BPs) under investigation with detailed information on the studied BPs, including characteristics related to Food Contact Chemical (FCC) typology and distinctions between IAS (intentionally added substances) and NIAS (non-intentionally added substances), Food Contact Material Number (FCM No.), relevant authorizations or compliance with current regulations, and specific migration limits (SML) in mg/kg, are presented in Table 1.

### 2.2. Sample Preparation

The analytical method for detecting BPs was developed using a rigorous protocol to ensure reliability and reproducibility. Glassware, including volumetric flasks, microsyringes, pipettes, and collection vials, was carefully cleaned with tap water, acetone, and methanol to prevent contamination. Glass containers were used for sample handling to avoid interference from plastic materials, particularly polycarbonate, which could release BPA. For the analysis of BPs in the bottled natural mineral water matrix, the extraction procedure involved subjecting 1 L of sample to SPE extraction using AFFINIMIP^®^ SPE cartridges specific for BPs (Polyintell, France). These cartridges were used according to the manufacturer’s protocol, including preconditioning, sample loading, washing to remove interferences, and elution steps to ensure high specificity and recovery of BPs. The SPE process was performed with a vacuum-based system equipped with flow regulators for precise control, along with a nitrogen flow evaporation system for eluate processing. Prior to processing, samples were spiked with an isotopically labeled standard. The eluate was evaporated under nitrogen to about 1 mL and then re-dissolved in a water/methanol solution before the analysis. Sample blanks, consisting of bottled natural mineral water free from analytes, were prepared to assess method specificity, control for contamination, and generate calibration curves.

To conduct a screening for potential BPs in PET/rPET, the sample preparation process followed the method described previously by Dreolin et al. [10]. Briefly, 0.4 g of the powdered sample was weighed into a quartz vessel, and 7.5 g of HFIP was added to ensure complete polymer dissolution. All samples, including the preforms (previously cut into smaller pieces of approximately 1 × 1 cm to facilitate processing), were cryomilled into a fine powder prior to HFIP dissolution. This procedure ensured a uniform particle size across all samples, minimizing potential variability in bisphenol extraction due to differences in particle size. The sample was sonicated for 3 h at 40 °C to achieve full polymer dissolution and to reduce the risk of analyte degradation. Subsequently, 8 mL of methanol (MeOH) was added. The sample was shaken for approximately 1 min and then stored at 4 °C for 1 h to allow polymer precipitation. The liquid fraction was placed into a centrifuge tube. The remaining precipitated polymer was rinsed twice with 1.0 mL of pure methanol, and the resulting washings were pooled with the initial liquid phase. The combined extract was centrifuged at 247× *g* for 10 min. Subsequently, the supernatant was retrieved and concentrated to a final volume of 2 mL by evaporation under a gentle nitrogen stream at ambient temperature.

### 2.3. BPs Analysis by UPLC-MS/MS

The quali- and quantitative analyses of BPs were carried out using a Thermo Scientific™ UltiMate™ 3000 (Thermo Fisher Scientific, Milan, Italy) Ultra High-Performance Liquid Chromatography (UHPLC) system coupled to a TSQ Fortis Triple Quadrupole Mass Spectrometer (MS). The analytes were separated using a Kinetex^®^ C18 column kept at a temperature of 40 °C. The mobile phase consisted of methanol (Phase A) and water (Phase B), both containing 0.045 mM ammonium hydroxide (NH_4_OH) solution. The injection volume for the UHPLC-MS/MS analysis was 20 µL.

### 2.4. Method Quality Assurance

The method was validated in compliance with the protocol established by the EURACHEM guidelines [25,26]. Following the guidelines mentioned above, the method validation focused on evaluating performance parameters such as the working range with linearity, detection thresholds (LODs), quantification thresholds (LOQs), precision, and reproducibility. The linearity criterion was considered fulfilled when the correlation coefficient (R^2^) was ≥0.990. For recovery assessments, both precision and reproducibility were regarded as acceptable if they fell within the 80–120% range. The data distribution normality was assessed using the Shapiro–Wilk test at a 95% confidence level, while potential outliers were detected and excluded using the Dixon and Grubbs tests [27].

### 2.5. Bottled Natural Mineral Water and PET/rPET Samples

To evaluate the method’s precision and accuracy, the samples analyzed included bottled natural mineral water (of the same brand, purchased from different local supermarkets; the brand name is withheld for privacy reasons), virgin PET (vPET) granules (Figure S1a), and recycled PET (rPET) granules (Figure S1b).

Alternatively, to carry out a screening for potential BPs in PET/Rpet, five distinct sample types, including granules, pellets, and preforms made from both PET and rPET, were randomly collected from a company based in the EU. The identities of the manufacturers are kept confidential due to a non-disclosure agreement. The sample types examined are illustrated in Figure S1.

## 3. Results and Discussion

### 3.1. Analytical Optimization

The method was optimized by adjusting the elution gradient to achieve optimal separation of the analytes. The specific gradient conditions are shown in Table S1. Regarding the MS acquisition, the method was performed using Multiple Reaction Monitoring (MRM) mode, achieved through Collision-Induced Dissociation (CID) of precursor ions in the collision cell. Characteristic product ions of the [M − H]^−^ molecule were monitored (see Table S2). The refined instrumental settings used during the analysis are listed in Table S3.

### 3.2. Method Performance Verification

#### 3.2.1. Linearity, LODs, and LOQs

Linearity was assessed by examining the relationship between varying concentrations of the analytes and their corresponding instrumental signals. Specifically, calibration solutions for eight BPs (BPA, BPB, BPS, BPF, BPAF, BPAP, BPZ, BPP) were prepared and analyzed using UPLC-MS/MS. For instance, for BPA and BPB, linear ranges were carried out by analyzing seven calibration points (0.10, 0.25, 0.50, 1.0, 2.5, 5.0, and 10.0 µg/L), and six calibration points (0.25, 0.50, 1.0, 2.5, 5.0, and 10.0 µg/L) were used for BPS, BPF, BPAF, BPAP, BPZ, and BPP. In Table 2, the correlation coefficients (R^2^), which fulfilled the required criteria (≥0.990) for all the tested analytes, the LODs and the LOQs were indicated. Particularly, for bottled natural mineral water, the LODs were in the range 0.030–0.075 µg/L, and the LOQs ranged from 0.10 µg/L to 0.25 µg/L. By contrast, for the PET and rPET matrices, the LODs were in the range 0.00030–0.00075 mg/kg, and the LOQs ranged from 0.0010 mg/kg to 0.0025 mg/kg.

#### 3.2.2. Repeatability and Accuracy

To evaluate the precision and accuracy of the analytical method and to prevent any contamination during the procedure, recovery tests were performed on spiked samples. Specifically, bottled natural mineral water, virgin PET (vPET) (Figure S1a), and recycled PET (rPET) granules (Figure S1b) were spiked at three different concentration levels and then subjected to the complete BPs extraction and instrumental analysis workflow. The results were assessed by calculating mean values, standard deviations (SD), relative standard deviations (RSD, %), and recovery rates (%Rec). Specifically, the recoveries were determined using Equation (1) [28]:

(1) $$\%Rec\;=\;\frac{\left(C_{spiked}-\;C_{ref}\right)}{C_{add}}\;\;\;\times 100$$

where *C_spiked_* (mg/kg) represents the amount of the analyte in the spiked sample, *C_ref_* (mg/kg) denotes the analyte concentration in the unspiked sample, and *C_add_* (mg/kg) refers to the nominal concentration of an analytical standard used for spiking. Specifically, the repeatability and accuracy were assessed for bottled natural mineral water at 0.1, 1, and 10 µg/L for BPA and BPB, and at 0.25, 1, and 10 µg/L for BPS, BPF, BPAF, BPAP, BPZ, BPP. For PET/rPET matrices, the repeatability and accuracy were assessed at 0.001, 0.010, and 0.100 mg/kg for BPA and BPB, and at 0.0025, 0.010, and 0.100 mg/kg for BPS, BPF, BPAF, BPAP, BPZ, and BPP. The results pertaining to the recoveries obtained for evaluating the repeatability of the method are detailed comprehensively in Table 3.

The repeatability of the method was assessed using relative standard deviation (RSD%) values for each analyte at three spiked concentration levels in bottled natural mineral water, PET, and rPET samples. The results demonstrate consistent performance, with all the RSD% values below 20%, indicating satisfactory repeatability across all matrices. As shown in Table 3, RSD% ranged from 2.0% to 13.1% for bottled natural mineral water, 1.9% to 12.3% for PET samples, and 0.5% to 6.9% for rPET samples. As expected in trace-level analysis, slightly higher variability was observed at the lowest spiked levels (e.g., 0.001 mg/kg or 0.0025 mg/kg). However, at higher concentrations (e.g., 0.01 mg/kg and 0.1 mg/kg), RSD% values significantly improved, frequently falling below 5%. These findings confirm the robustness of the method and its suitability for the precise quantification of BPs in the tested sample matrices.

**Figure 2:**
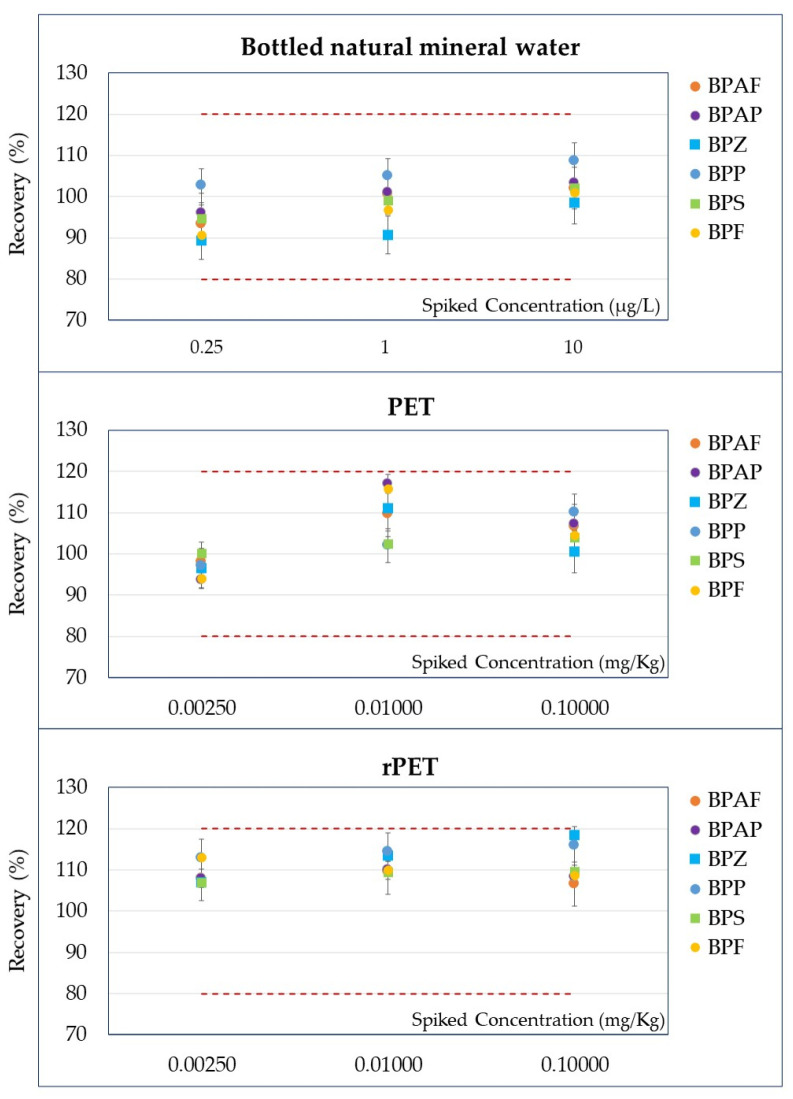
Percentage recoveries (on the *y*-axis) obtained for BPAF, BPAP, BPZ, BPP, BPS, and BPF at three spiked levels (on the *x*-axis) with the RSDs as error bars in bottled natural mineral water, in virgin PET samples, and in rPET samples.

**Figure 3:**
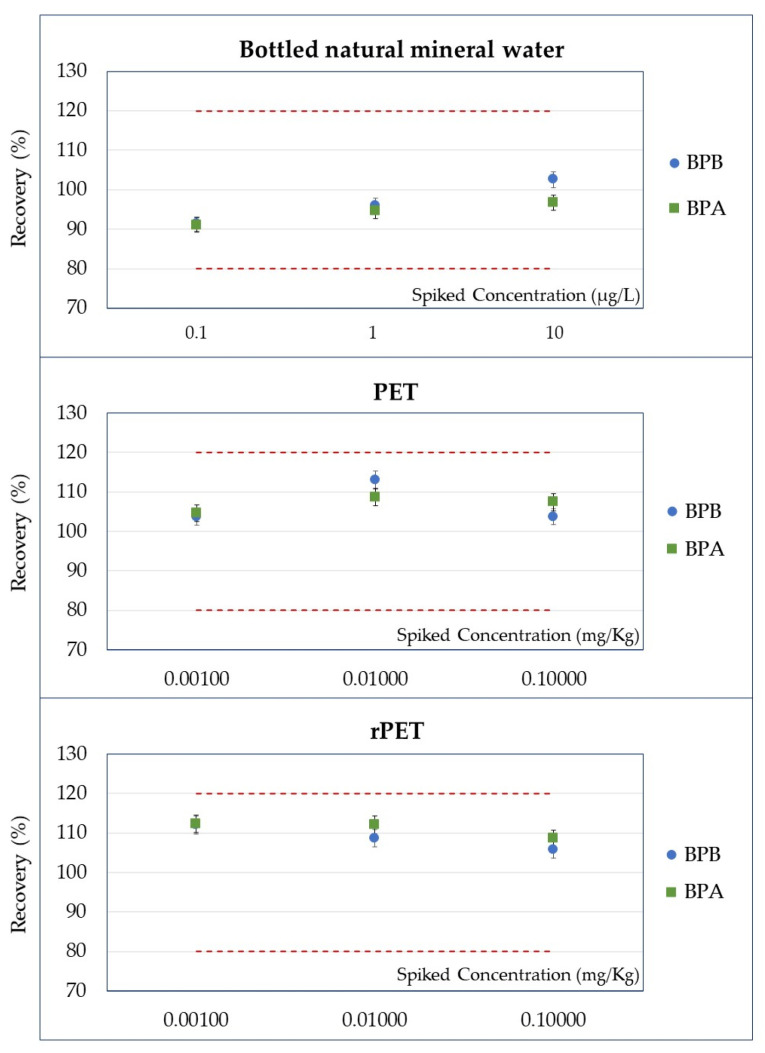
Percentage recoveries (on the *y*-axis) obtained for BPB and BPA at three spiked levels (on the *x*-axis) with the RSDs as error bars in bottled natural mineral water, in vPET samples, and in rPET samples.

The method’s accuracy was evaluated by comparing the mean analyte concentrations measured in bottled natural mineral water, PET, and rPET samples at three spiked levels (low, medium, and high) with their corresponding nominal values. Across all matrices and BPs, the measured concentrations closely matched the spiked levels, with deviations consistently within acceptable analytical ranges. Even at the lowest spiked levels (e.g., 0.001 mg/kg or 0.0025 mg/kg), the mean values demonstrated good agreement with the nominal concentrations, reflecting the method’s capability for reliable detection of trace-level analytes. At higher spiked levels (e.g., 0.01 mg/kg and 0.1 mg/kg), accuracy further improved, with measured concentrations deviating minimally from the target values. Specifically, mean recoveries for all studied BPs in bottled natural mineral water ranged from 89% (observed for BPZ at the lowest validation level) to 109% (measured for BPP at the highest validation level), demonstrating the method’s reliability and precision. In contrast, recoveries in PET samples ranged from 94% (recorded for BPAP at the lowest validation level) to 117% (at the intermediate validation level for the same analyte). For rPET samples, recoveries varied between 106% (detected for BPB at the spiked level of 0.100 mg/kg) and 118% (measured for BPZ at the highest validation level). The percentage recoveries for BPAF, BPAP, BPZ, BPP, BPS, and BPF (Figure 2) and BPB and BPA (Figure 3) visually demonstrate these results, with RSD values shown as error bars.

This graphical data further confirms the method’s accuracy across all matrices, supporting its robustness for routine analytical applications. The results obtained from the method validation confirm the robustness, reliability, and applicability of the analytical method for BPs identification and quantification in bottled natural mineral water, PET, and rPET matrices. The linearity assessment demonstrated satisfactory performance, with correlation coefficients (R^2^) meeting the acceptance criteria of ≥0.990, ensuring reliable and accurate quantification across the tested range. Recovery values for all BPs across all spiking levels met the acceptance criteria of 80–120%, highlighting the method’s strong accuracy and precision. Repeatability was evidenced by RSD% values, which remained predominantly below 10% at higher spiking levels, with slightly higher variability observed only at trace concentrations. In summary, these findings validate the method’s reliability, establishing it as a robust tool for routine BP analysis across diverse matrices.

### 3.3. BPs in vPET and rPET Samples

**Figure 4:**
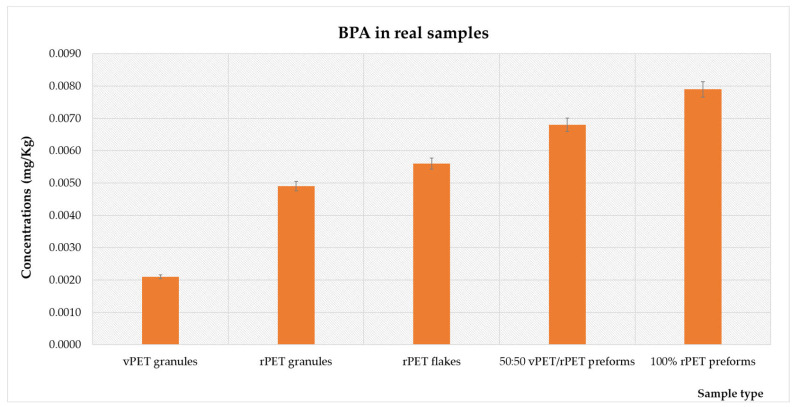
Concentrations (mg/kg) of BPA assessed in vPET and rPET samples.

The results obtained from the analysis of granule, flake, and preform samples for the identification and quantification of the eight BPs are illustrated in Figure 4.

As is clear from the figure, only BPA was found in quantifiable concentrations in the analyzed samples. The concentrations of all other investigated bisphenols were below the limit of quantification. Specifically, for BPA, the average concentration observed (calculated as the mean of the concentrations across all matrices ± standard deviation) was 0.0055 ± 0.0022 mg/kg. In detail, the average concentration found in the vPET matrix was 0.0021 ± 0.0007 mg/kg, while in rPET granules, the concentration was 0.0049 ± 0.0011 mg/kg. For rPET flakes, the concentration measured was 0.0056 ± 0.0015 mg/kg. The average concentration in 50:50 vPET/rPET preforms was 0.0068 ± 0.0012 mg/kg, and in 100% rPET preforms, it was 0.0079 ± 0.0016 mg/kg. These results indicate a clear increasing trend in BPA concentrations as the transition occurs from vPET to rPET, with a more pronounced rise in concentrations as the proportion of recycled material increases. It is also worth noting that the levels for other bisphenols were below the detection limits, suggesting minimal presence or complete absence in the analyzed samples.

Although BPA is not intentionally used in the production of PET, its presence or migration from PET bottles cannot be ruled out [29]. Nevertheless, a review highlighted numerous studies reporting the migration of BPA from PET bottles into beverages [2]. Particularly, the presence of BPA in vPET and rPET matrices, including granules, flakes, and preforms, can be attributed to several factors. In PET, BPA may originate from residual monomers or additives used during polymerization [30]. In fact, it is well known that BPA is used to enhance the quality of plastic materials, including PET, contributing to their performance and suitability for various applications in consumer and industrial products [31]. In rPET, trace levels of contamination could be introduced through the rPET content, as BPA is commonly found everywhere [29]. Furthermore, BPA contamination can result from the recycling process, where residual BPA from previously used materials or from additives incorporated during recycling can persist [10]. Additionally, the widespread use of BPA in various industrial applications, including its presence in polycarbonate plastics and epoxy resins, contributes to its prevalence in recycled materials [32].

However, although it is widely acknowledged that BPA should not be present in PET, studies currently available in the literature report its occurrence [10,13,29]. Nevertheless, research evaluating and quantifying this analyte in PET and rPET matrices remains limited, as most studies have focused on assessing BPA migration from FCMs. Additionally, nearly all investigations have been conducted on finished bottles, overlooking granules, flakes, and preforms. This has led to a significant gap in understanding the entire production and recycling process of PET, including identifying potential sources of contamination and the stages at which they may arise during the process. Furthermore, the majority of research has focused exclusively on BPA, neglecting the potential presence of other BPs, which are increasingly emerging as alternative substitutes for BPA. Notably, for these alternative BPs, no regulatory limits have been established to date, unlike BPA, for which the European Food Safety Authority (EFSA) in 2023 proposed a new tolerable daily intake (TDI) of 0.0002 µg/kg body weight per day. This emphasizes the critical need for monitoring and controlling BPA levels in rPET products [29].

In this context, a recent study tracked BPA levels in PET bottles made with rPET, used for storing drinking water and soft drinks over a five-year period (2019 to 2023) [29]. BPA concentrations were measured using LC-MS. Their findings showed that annual mean BPA concentrations ranged from 0.715 to 4.14 mg/kg. A significant rise in BPA levels was observed starting in 2021, likely due to a higher proportion of post-consumer recycled material in PET bottles [29]. Similarly, another study [10] investigated BPA levels in 23 PET samples, including ten virgin PET, nine 100% recycled PET, and four with varying percentages of recycled content, using LC-MS/MS. Their study reported BPA concentrations ranging from 0.025 to 0.432 mg/kg in virgin PET, while recycled PET exhibited higher levels, ranging from 0.394 to 10.120 mg/kg [10]. The results of the present study align with these findings, confirming that BPA concentrations tend to increase as the proportion of recycled content in PET rises. The increasing BPA concentrations observed from vPET to rPET matrices may be due to BPA accumulation during recycling, as incomplete contaminant removal leads to higher levels in the final rPET products. This aligns with Dreolin et al.’s conclusion that BPA concentration exceeding 0.500 mg/kg PET could indicate recycled material presence, given that PET is only exposed to BPA during recycling [10]. However, our research is consistent with other similar studies [33] findings of increasing BPA concentrations with higher recycled content percentages. Additionally, Steimel’s study also pointed to potential BPA contamination sources in the recycling process, including printing inks, bottle caps, environmental contamination, or improper sorting [12,34]. While Fan et al. reported BPA concentrations in Chinese market PET bottles ranging from 0.0641 ± 0.0058 mg/kg to 0.0377 ± 0.0057 mg/kg, measured by HPLC after extraction of ethanol with ultrasonication, their study did not clarify if these PET bottles contained recycled material [35]. Finally, a study [13], which analysed BPA, BPS, and BPF in commercially available recycled plastics including PET using UHPLC-MS/MS, found BPA concentrations of 0.00145 ± 0.00005 mg/kg in vPET, compared to 0.251 ± 0.083 mg/kg, 0.326 ± 0.080 mg/kg, and 2.35 ± 0.08 mg/kg in rPET samples. Their study also reported low BPs (0.00015 ± 0.00003 mg/kg) concentrations in vPET (<LOQ to 0.00061 ± 0.00009 mg/kg in rPET), as well as BPF (0.00059 ± 0.00007 mg/kg in vPET, <LOQ and 0.00138 ± 0.00025 mg/kg in rPET) concentrations, suggesting generally low bisphenol contamination in virgin plastics, with higher levels in recycled materials. The study further highlights the complexity of bisphenol contamination in plastics and showed that washing processes can effectively reduce bisphenol levels in recycled plastics, although one PET sample showed an increase in BPA after washing. Similarly, Nuygen et al., using HSGC-MS, reported BPA concentrations of 0.24 ± 0.04 mg/kg and 0.38 ± 0.02 mg/kg in washed colored and transparent PET flakes, respectively, suggesting a potential link between plastic color and bisphenol concentrations. These findings emphasize the multifaceted nature of bisphenol contamination in PET and the need for more research to effectively address it [36].

In Italy, Legislative Decree 18/2023, implementing EU Directive 2020/2184, establishes a maximum allowable concentration of 2.5 µg/L for BPA in drinking water and introduces a risk-based framework requiring systematic monitoring, safety planning, and public reporting [37]. The European Union adopts this limit to ensure consistent standards across Member States. By contrast, the United States currently does not set a federal maximum contaminant level for BPA in drinking water, although the EPA may issue non-binding health advisories. These provisions apply exclusively to BPA and do not extend to other bisphenols examined in this study. Regarding food contact materials, under EU Regulation 10/2011, BPA migration from PET was previously limited to 50 µg/kg, while from 2025 its use will be entirely prohibited except in cases of negligible migration. It should be noted that for food contact materials, no maximum allowable concentrations of BPA in the material itself are currently defined; regulations refer solely to migration into food. In the United States, the FDA does not specify explicit migration limits for BPA in PET, although its use is prohibited in baby bottles and otherwise regulated through general safety assessments. In light of these frameworks, the calibration range employed in this study (0.10–10 µg/L, LOQ 0.10 µg/L) is consistent with current legislation, fully encompassing the legal limit of 2.5 µg/L for bottled natural mineral water. Overall, this regulatory landscape reflects stricter control in Italy and the EU compared to the U.S. approach, both for drinking water and food-contact materials, and emphasizes that these limits concern BPA only, not the other bisphenols analyzed in this study.

The findings of this study, alongside previous research, highlight the urgent need for stringent monitoring and control measures during the recycling process to minimize BP contamination in rPET products, ensuring their safety for food contact applications. To address this challenge effectively, it is critical for the development of highly sensitive and accurate analytical methods capable of detecting NIAS, including BPs, in food-contact materials. Such methodologies are essential not only for identifying contamination sources throughout the recycling process but also for providing a comprehensive assessment of the presence of bisphenol analogs. As these compounds, often used as substitutes for BPA in the PET recycling, become increasingly prevalent and remain largely unregulated, their inclusion in monitoring protocols is crucial for safeguarding consumer health.

## 4. Conclusions

This study effectively developed and validated a reliable and sensitive method for detecting and quantifying BPs in bottled natural mineral water, PET, and rPET matrices, including granules, flakes, and preforms. The method demonstrated excellent linearity, precision, and repeatability, meeting rigorous analytical standards. The results confirmed that while BPA was quantifiable in all tested matrices, other BPs remained below the limit of quantification, underlining the specificity and effectiveness of the developed method. A key finding of this study is the increase in BPA concentrations as the proportion of recycled content in PET rises, with the highest concentrations identified in 100% rPET preforms. The presence of BPA in recycled materials emphasizes the need for strict quality control measures during the PET recycling process. Additionally, this study revealed that while virgin PET exhibited minimal bisphenol contamination, recycled materials presented significantly higher concentrations, likely due to cumulative exposure during their lifecycle and contamination from external sources in the recycling.

It should be noted that the rPET samples analyzed in this study originated from a single mechanical recycling process. As rPET properties and potential bisphenol content may vary depending on the recycling route, the results are specific to this stream. Future studies will include samples from multiple recycling processes to assess the generality of the findings. Moreover, this study highlights the significance of monitoring bisphenol analogs, which serve as unregulated substitutes for BPA in plastics, posing potential food safety risks. The development of advanced detection methods is essential for understanding contamination in PET production and recycling. The validated method presented here provides a reliable tool for future studies on bisphenol contamination, and emphasizes the need for ongoing research to ensure food safety and inform regulatory efforts supporting sustainable PET recycling.

## Figures and Tables

**Table 1:** List of analytes (BPs) under examination, with IUPAC and common names, abbreviations, molecular formula, Food Contact Material Number (FCM No), the necessary authorization or adherence to applicable regulations, and the specific migration limits (SML) expressed in mg/kg.

Analyte (Common/IUPAC Name)	Abbreviation	Molecular Formula	FCM No ^a^	Authorized ^b^	SML ^b^ (mg/kg)
Bisphenol A (4-[2-(4-hydroxyphenyl)propan-2-yl]phenol)	BPA	C_15_H_16_O_2_	151	Not Authorized *	0.05
Bisphenol S (4-(4-hydroxyphenyl)sulfonylphenol)	BPS	C_12_H_10_O_4_S	154	Not Authorized *	0.05
Bisphenol F (4-[(4-hydroxyphenyl)methyl]phenol)	BPF	C_13_H_12_O	90	Not Authorized *	60
Bisphenol B (4-[2-(4-hydroxyphenyl)butan-2-yl]phenol)	BPB	C_16_H_18_O_2_	-	Not Authorized *	-
Bisphenol AF (4-[1,1,1,3,3,3-hexafluoro-2-(4-hydroxyphenyl)propan-2-yl]phenol)	BPAF	C_15_H_10_F_6_O_2_	-	Not Authorized *	-
Bisphenol AP (4-[1-(4-hydroxyphenyl)-1-phenylethyl]phenol)	BPAP	C_20_H_18_O_2_	-	Not Authorized *	-
Bisphenol Z (4-[1-(4-hydroxyphenyl)cyclohexyl]phenol)	BPZ	C_18_H_20_O_2_	-	Not Authorized *	-
Bisphenol P (4-[2-[4-[2-(4-hydroxyphenyl)propan-2-yl]phenyl]propan-2-yl]phenol)	BPP	C_24_H_26_O_2_	-	Not Authorized *	-

* These BPs are not listed in Annex I of Commission Regulation (EU) 2020/1245 and are not approved for use in PET FCMs [23]. ^a^ Commission Staff Working Document Evaluation of The Legislation on Food Contact Materials—Regulation (EC) No 1935/2004 [24]. ^b^ Commission Regulation (EU) 2020/1245 of 2 September 2020 amending and correcting Regulation (EU) No 10/2011 on plastic materials and articles intended to come into contact with food [23].

**Table 2:** List of studied analytes, the range of calibration curves, with equations and correlation coefficients (R^2^), the LODs, and the LOQs.

Analyte		Calibration Range (µg/L)	Calibration Equation	R^2^	Bottled Natural Mineral Water		PET/rPET	
					LOD (µg/L)	LOQ (µg/L)	LOD (mg/kg)	LOQ (mg/kg)
BPs	BPA	0.10–10	y = 0.210x + 0.025	0.993	0.030	0.10	0.00030	0.0010
	BPS	0.25–10	y = 0.074x + 0.015	0.991	0.075	0.25	0.00075	0.0025
	BPF	0.25–10	y = 0.132x + 0.010	0.992	0.075	0.25	0.00075	0.0025
	BPB	0.10–10	y = 0.165x + 0.020	0.992	0.030	0.10	0.00030	0.0010
	BPAF	0.25–10	y = 0.105x + 0.020	0.993	0.075	0.25	0.00075	0.0025
	BPAP	0.25–10	y = 0.095x + 0.018	0.990	0.075	0.25	0.00075	0.0025
	BPZ	0.25–10	y = 0.160x + 0.022	0.994	0.075	0.25	0.00075	0.0025
	BPP	0.25–10	y = 0.100x + 0.012	0.992	0.075	0.25	0.00075	0.0025

**Table 3:** Method validation outcomes for repeatability, including the analyte concentrations (expressed as mean values) with SD and RSD% for bottled natural mineral water (µg/L), PET samples (mg/kg), and rPET samples (mg/kg) across three spiked levels.

	Analyte	C_spiked_ (µg/L)	Bottled Natural Mineral Water		C_spiked_ (mg/kg)	PET		rPET	
			Mean ± SD (µg/L)	RSD (%)		Mean ± SD (mg/kg)	RSD (%)	Mean ± SD (mg/kg)	RSD (%)
BPs	BPA	0.1	0.091 ± 0.006	6.6	0.001	0.0010 ± 0.0001	8.1	0.0011 ± 0.0002	3.7
		1	0.95 ± 0.09	9.1	0.01	0.0109 ± 0.0006	5.2	0.0112 ± 0.0003	2.7
		10	9.67 ± 0.19	2.0	0.1	0.1074 ± 0.0041	3.8	0.1086 ± 0.0042	3.9
	BPS	0.25	0.24 ± 0.03	12.9	0.0025	0.0025 ± 0.0002	6.9	0.0027 ± 0.0001	4.0
		1	0.99 ± 0.10	9.7	0.01	0.0102 ± 0.0008	7.8	0.0109 ± 0.0003	2.3
		10	10.20 ± 0.34	3.4	0.1	0.1039 ± 0.0027	2.6	0.1095 ± 0.0006	0.5
	BPF	0.25	0.23 ± 0.02	6.7	0.0025	0.0024 ± 0.0003	11.8	0.0028 ± 0.0001	3.3
		1	0.97 ± 0.05	4.9	0.01	0.0118 ± 0.0008	6.5	0.0110 ± 0.0004	3.3
		10	10.09 ± 0.28	2.8	0.1	0.1045 ± 0.0020	1.9	0.1086 ± 0.0021	1.9
	BPB	0.1	0.09 ± 0.01	7.1	0.001	0.0010 ± 0.0001	9.8	0.0011 ± 0.0000	3.9
		1	0.96 ± 0.07	7.3	0.01	0.0113 ± 0.0006	5.5	0.0109 ± 0.0005	4.1
		10	10.26 ± 0.59	5.7	0.1	0.1037 ± 0.0046	4.5	0.1058 ± 0.0026	2.4
	BPAF	0.25	0.23 ± 0.03	13.1	0.0025	0.0025 ± 0.0002	9.4	0.0027 ± 0.0001	4.3
		1	1.01 ± 0.09	8.7	0.01	0.0110 ± 0.0005	4.3	0.0110 ± 0.0005	4.3
		10	10.21 ± 0.57	5.6	0.1	0.1066 ± 0.0036	3.3	0.1066 ± 0.0036	3.3
	BPAP	0.25	0.24 ± 0.03	11.0	0.0025	0.0023 ± 0.0003	12.3	0.0027 ± 0.0002	6.8
		1	1.01 ± 0.09	8.5	0.01	0.0117 ± 0.0004	3.7	0.0110 ± 0.0008	6.9
		10	10.32 ± 0.26	2.5	0.1	0.1072 ± 0.0029	2.7	0.1083 ± 0.0022	2.0
	BPZ	0.25	0.22 ± 0.01	5.2	0.0025	0.0024 ± 0.0002	10.2	0.0027 ± 0.0001	4.0
		1	0.91 ± 0.05	5.2	0.01	0.0111 ± 0.0007	6.3	0.0113 ± 0.0004	3.3
		10	9.84 ± 0.33	3.3	0.1	0.1005 ± 0.0027	2.7	0.1183 ± 0.0013	1.1
	BPP	0.25	0.26 ± 0.02	8.1	0.0025	0.0024 ± 0.0002	8.1	0.0028 ± 0.0001	3.3
		1	1.05 ± 0.10	9.7	0.01	0.0102 ± 0.0010	9.8	0.0114 ± 0.0004	3.3
		10	10.87 ± 0.24	2.2	0.1	0.1100 ± 0.0030	2.7	0.1159 ± 0.0044	3.8

## Data Availability

The original contributions presented in the study are included in the article/Supplementary Materials; further inquiries can be directed to the corresponding author.

## Supplementary Materials

The following supporting information can be downloaded at: https://www.mdpi.com/article/10.3390/foods14172968/s1, Figure S1: (a) Virgin PET (vPET) granules; (b) recycled PET (rPET) granules; (c) rPET flakes; (d) preform 50% vPET/50% rPET; (e) preform 100% rPET; Table S1: Gradient elution program; Table S2: Mass spectrometry transition for acquisition in MRM mode; Table S3: List of studied analytes, the range of calibration curves, with equations and correlation coefficients (R2), the LODs, and the LOQs.
