# Supplementary material for: Optimized Method for Quantifying Bisphenols in Bottled Water and PET/rPET Matrices
Source: Foods. 2025 Aug 26;14(17):2968. doi: 10.3390/foods14172968 (PMC12428101; doi:10.3390/foods14172968)
Supplement: Supplementary file 1 [file foods-14-02968-s001.zip › foods-3817448-supplementary.pdf]

Supplementary Material

Optimized Method for Quantifying Bisphenols in Bottled Water and PET/rPET Matrices

Fabiana Di Duca [1,*], Paolo Montuori [1], Elvira De Rosa [1,2], Immacolata Russo [3], Raffaele Palladino [1,4,5] Stefano Scippa [1], Giuseppe Dadà [6], Maria Triassi [1,4], Sergi Díez [7]

[1] Department of Public Health, University "Federico II", Via Sergio Pansini 5, 80131 Naples, Italy
[2] Department of Human Sciences and Quality of Life Promotion, San Raffaele University, 00166 Rome, Italy
[3] Department of Public Health, University Hospital of Naples "Federico II", Via Sergio Pansini 5, 80131 Naples, Italy
[4] Interdepartmental Research Center in Healthcare Management and Innovation in Healthcare (CIRMIS), Via Sergio Pansini 5, 80131, Naples, Italy
[5] Department of Primary Care and Public Health, School of Public Health, Imperial College, London SW7 2AZ, UK
[6] CORIPET-Consorzio Volontario per Riciclo del PET, Via S. Maurilio 23, 20123 Milan, Italy
[7] CSIC-Instituto de Diagnostico Ambiental y Estudios del Agua (IDAEA), C/Jordi Girona, 18-26, E-08034 Barcelona, Spain
* Correspondence: fabianadiduca91@gmail.com

Figure S1. (**a**) Virgin PET (vPET) granules; (**b**) Recycled PET (rPET) granules; (**c**) rPET flakes; (**d**) Preform 50% vPET/50% rPET; (**e**) Preform 100% rPET.

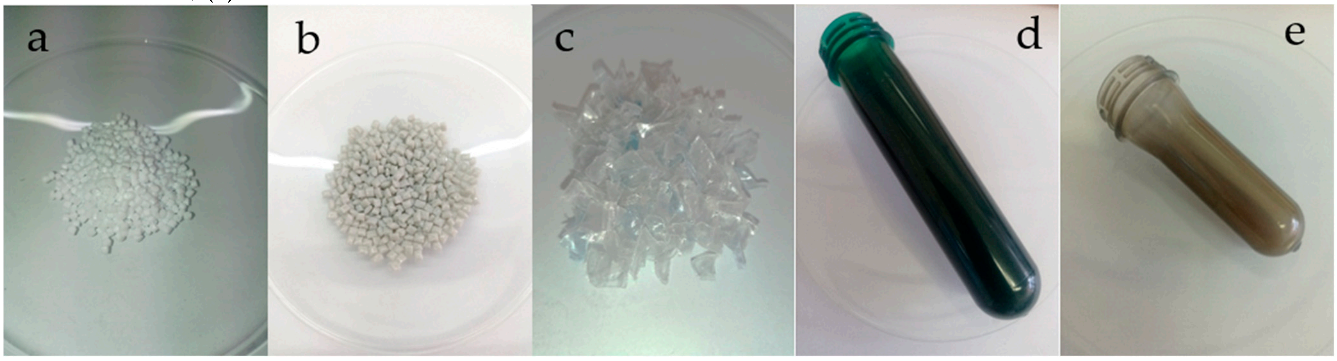

Table S1. Gradient elution profile for chromatographic analysis (%B = water with 0.045 mM NH_4OH in the mobile phase).

Time	Flow (μL/min)	%B
0.00	150	50
2.00	150	50
8.00	150	90
10.00	150	90
15.00	150	50

Table S2. Mass spectrometry transition for acquisition in MRM mode.

Analyte	Molecular Weight (g/mol)	Precursor Ion [m/z] $[M\text{-}H]^-$	Product Ion [m/z]
BPA	228.29	227.2	211
			133
BPS	250.27	249.0	108
			92
BPF	200.23	199.1	93
			105
BPB	242.32	241.1	212
			225
BPAF	336.23	335.1	265
			197
BPAP	290.36	289.0	274
			195
BPZ	268.35	267.0	223
			173
BPP	346.50	345.2	330
			133
BPA_{d16}	244.38	241.0	142
			222

Table S3. LC/MS conditions: global and scan parameters.

**Global Parameters**			
Method Duration	15 min		
**Ion source Properties**			
Ion Source Type	H-ESI	Sheath Gas (Arb)	35
Spray Voltage	Static	Aux Gas (Arb)	7
Positive Ion (V)	3500	Sweep Gas (Arb)	0
Negative Ion (V)	2500	Ion transfer tube Temp (°C)	300
Current LC Flow (ug/min)	300	Vaporizer Temp (°C)	275
**Scan Parameters**			
Polarity	Negative	Source Fragmentation	5
Use Cycle Time	✓	Chromatographic Peak Width (sec)	6
Cycle Time (sec)	0.8	Use Chromatographic Filter	✓
Use Calibrated Tube Lens	✓	Use Retention time reference	×
Q1 Resolution (FWHM)	1.2	Display Retention Time	✓
Q3 Resolution (FWHM)	1.2	Use quan ion	×
CID Gas (mTorr)	1.5	Show visualization	×
